# Silicon-induced changes in plant volatiles reduce attractiveness of wheat to the bird cherry-oat aphid *Rhopalosiphum padi* and attract the parasitoid *Lysiphlebus testaceipes*

**DOI:** 10.1371/journal.pone.0231005

**Published:** 2020-04-03

**Authors:** Reinaldo Silva de Oliveira, Maria Fernanda G. V. Peñaflor, Felipe G. Gonçalves, Marcus Vinicius Sampaio, Ana Paula Korndörfer, Weliton D. Silva, José Maurício S. Bento

**Affiliations:** 1 Instituto de Ciências Agrárias, Universidade Federal de Uberlândia, Uberlândia, MG, Brazil; 2 Departamento de Entomologia, Universidade Federal de Lavras, Lavras, MG, Brazil; 3 Departamento de Entomologia e Acarologia, Universidade de São Paulo, Escola Superior de Agricultura “Luiz de Queiroz”, Piracicaba, SP, Brazil; Zhejiang University, CHINA

## Abstract

Silicon (Si) supplementation is well-known for enhancing plant resistance to insect pests, however, only recently studies revealed that Si accumulation in the plant not only confers a mechanical barrier to insect feeding, but also primes jasmonic acid-dependent defenses. Here, we examined whether Si supplementation alters wheat volatile emissions that influence the bird cherry-oat aphid (*Rhopalosiphum padi*) olfactory preference and the aphid parasitoid *Lysiphlebus testaceipes*. Even though Si accumulation in wheat did not impact aphid performance, we found that *R*. *padi* preferred constitutive volatiles from–Si wheat over those emitted by +Si wheat plants. In Y-tube olfactometer bioassays, the parasitoid was attracted to volatiles from +Si uninfested wheat, but not to those from–Si uninfested wheat. +Si and–Si aphid-infested plants released equally attractive blends to the aphid parasitoid; however, wasps were unable to distinguish +Si uninfested plant odors from those of aphid-infested treatments. GC-MS analyses revealed that +Si uninfested wheat plants emitted increased amounts of a single compound, geranyl acetone, compared to -Si uninfested wheat, but similar to those emitted by aphid-infested treatments. By contrast, Si supplementation in wheat did not alter composition of aphid-induced plant volatiles. Our results show that changes in wheat volatile blend induced by Si accumulation mediate the non-preference behavior of the bird cherry-oat aphid and the attraction of its parasitoid *L*. *testaceipes*. Conversely to the literature, Si supplementation by itself seems to work as an elicitor of induced defenses in wheat, and not as a priming agent.

## Introduction

The role of Silicon (Si) in plant nutrition is controversial [1–3]. Nevertheless, interest on Si supplementation as an agronomic practice has been growing because it generally promotes increased crop yield [4], especially of those that are Si-accumulators plants, such as Poaceae [5]. Si is taken up in the water-soluble form of Si(OH)_4_ from the soil and translocated to the shoot, where it precipitates as amorphous silica forming phytoliths [3, 6]. The deposition of Si in the plant tissues often enhances plant growth [7] as well as resistance against abiotic and biotic stresses, such as drought, heat, salinity, toxicity by heavy metals, pathogen infections and herbivory by arthropods [8–10].

Numerous studies have demonstrated that accumulation of Si in the shoots of crop grasses induces resistance against a wide range of herbivorous arthropods of different feeding guilds, such as leaf feeders [11], sap-sucking insects [12], stalk borers [13] and piercing-sucking [14]. Feeding on Si-supplemented plants, in general, increases insect development time [15] and reduces weight gain [16], fecundity [17] and survival [11]. It has been long thought that Si conferred resistance against herbivores due to the increased leaf abrasiveness that served as a mechanical barrier to herbivory [18], thereby reducing food quality to herbivores as well as increasing their mandible wear [19]. However, accumulating evidence supports that cell silicification is not the only underlying mechanism of Si-induced resistance against herbivores, but biochemical changes are also involved [20]. For example, Si supplementation promotes higher activity levels of defense-related enzymes particularly in plants following insect attack [11, 21], indicating a priming effect. Ye et al. [22] elucidated that jasmonic acid-signaling pathway, which plays a central role in activating antiherbivore induced defenses [23], is involved in Si-induced resistance and that Si enhances the accumulation of jasmonic acid (JA) levels following herbivory, confirming the priming effect.

Elevated JA is usually involved in the activation of induced plant defenses against herbivores, but the interaction between JA, salicylic acid and ethylene is important in fine-tuning of plant defenses [24]. Herbivore-induced defenses include synthesis of toxic and deterring chemicals to herbivores as well as release of volatile organic compounds, called herbivore-induced plant volatiles (HIPVs), which recruit herbivore natural enemies [25, 26]. Therefore, given the priming effect of Si on JA-signaling pathway, treatment with Si may not only result in enhanced direct defenses against herbivores, as a vast number of studies show [27], but also indirect ones through emission of attractive plant volatile blends to natural enemies.

Recent reviews have pointed out the potential of Si as an elicitor of plant direct and indirect resistance [20, 28]. However, to the best of our knowledge, just recently a study has demonstrated that Si deposition enhances recruitment of herbivore natural enemies through emission of HIPVs [29]. The authors revealed that Si-supplemented plants emitted a HIPV blend more attractive to the third trophic level than that emitted by non-supplemented plants, resulting in elevated predation in Si-supplemented plants in the field. The few studies that have focused on the effects of Si plant supplementation on predation/parasitism rates [30, 31] or natural enemy recruitment [32] did not examine whether changes on HIPVs mediated the tritrophic interaction.

Because of the pest status of aphids on cereal crops, effects of Si supplementation in wheat against aphids have been well studied. In general, Si-supplemented wheat is a host of poor quality and therefore deleterious to their development and reproduction [33–35]. Aphids avoid colonizing Si-supplemented wheat plants [21, 35, 36], however, once in contact with the plant, they usually spend the same time for stylet penetration in Si-supplemented and non-Si supplemented wheat plants [34, 37], indicating that the supposed physical barrier provided by the deposition of Si in the epidermis does not play a role in the Si-induced resistance against aphids. Findings by Gomes et al. [21] also support the hypothesis that resistance of Si-supplemented wheat against aphids is mediated by the activation of induced chemical defenses. Nevertheless, no study has proved, hitherto, that deposition of Si in wheat induces chemical defenses against aphids.

The bird cherry-oat aphid *Rhopalosiphum padi* (L.) (Hemiptera: Aphididae) is one of the most important pests of wheat worldwide, especially because it transmits the pathogen *barley yellow dwarf virus* (BYDV) [38]. Losses in grain yield in *R*. *padi*-infested wheat areas can reach 58% due to aphid feeding and BYDV infection [39]. Although parasitoids can provide a significant control of cereal aphid populations [40], chemical control is widely employed to suppress population of *R*. *padi* in cereal crops, causing undesirable effects, such as development of resistance [41]. In this context, given the above-mentioned promising results as a tactic to control aphids in wheat, Si supplementation offers a promise for aphid management in order to rely less on pesticides.

Here, we investigated whether Si supplementation induces volatile chemical defenses in wheat against *R*. *padi*. We specifically studied the olfactory response of the aphid *R*. *padi* and its parasitoid *Lysiphlebus testaceipes* (Cresson) (Hymenoptera: Braconidae: Aphidiinae), an important biological control agent of *R*. *padi* in wheat [42], to Si-supplemented and non-supplemented wheat plant volatile emissions. Previous studies have demonstrated that Si accumulation in grasses, including wheat, confers a non-preference type of resistance to aphids [35, 43, 44], but the underlying mechanism has yet to be investigated. Therefore, our study addressed whether the non-preference of Si-supplemented wheat to *R*. *padi* is influenced by changes on the plant volatile emission; and investigated the outcomes of Si supplementation on tritrophic interactions, a relevant but poorly studied effect.

## Materials and methods

### Plants

Wheat (*Triticum aestivum* cv. BRS 254) was grown from seeds on a Typic Quartzipsamment soil [45] (81% sand, 5% silt and 14% clay) with a low Si content (1.2 mg.L^-1^ of soil). This naturally low Si level provided the opportunity to assess the effect of the addition of further Si to the soil, according to Korndörfer et al. [46]. Calcium carbonate (970 kg.ha^-1^) and magnesium carbonate (380 kg.ha^-1^) were added to the soil, which was kept moist and incubated in plastic bags (up to 30 kg of soil) for 30 d. Thereafter, the soil was supplemented with ammonium sulfate (equivalent to 80 kg. ha^-1^ of N), superphosphate (80 kg.ha^-1^ of P_2_O_5_), potassium chloride (50 kg.ha^-1^ of K_2_O) and the mix of micronutrients FTE BR12 (100 kg.ha^-1^). Three seeds of wheat were sown in a plastic pot with 300 ml of the soil and, at the phenological stage Z10 [47], only one seedling was left per pot. Plants used in all assays were cultivated in an insect-free greenhouse under natural light and no temperature control (Piracicaba, SP, Brazil). Assays were conducted with plants at stage Z13 (three leaves unfolded), or approximately 18–25 d after emergence.

### Si supplementation and analysis

Each pot with wheat seedlings (+Si wheat plants) was supplemented once at the growth stage Z07 (coleoptiles emerged from caryopsis) with 0.350 g of ground silica gel (SiO_2_, 5.7% available Si) diluted in 15 ml of water, equivalent to 300 kg.ha^-1^ of Si. Control plants (-Si wheat plants), at the same time, received the same volume of water, but no Si. To measure leaf Si content, leaves of six plants, representing a replicate, were cut from -Si wheat and +Si wheat at stage Z13 (three leaves unfolded) following the method described in Korndörfer [48]. Ten replicates were performed to assess the amount of Si deposited in wheat leaves.

### Insects

Hundreds of the bird cherry-oat aphid and the parasitoid *L*. *testaceipes* were collected from wheat plots (*T*. *aestivum* cv. BRS 254) grown in an infested greenhouse of the Federal University of Uberlândia (UFU, Uberlândia, MG, Brazil), and taken to the laboratory to establish the insect rearing. The aphid population was maintained feeding on 20-day-old wheat seedlings inside Plexiglas cages (60 × 30 × 30 cm). Wheat plants were replaced every week. A separate *R*. *padi* colony, in the same conditions described above, was initially infested with approximately 10 1- to 2-day-old mated *L*. *testaceipes* females. After 5–7 d, the aphid colony was inspected for parasitized aphids (mummies), which were collected and individualized in 2-ml plastic tubes with a drop of 50% solution of honey in water deposited in the inner wall. Adult parasitoids were separated by sex and paired (1 female:1 male) before releasing them into acrylic cages (60 × 30 × 30 cm) containing *R*. *padi*-infested wheat. This procedure was repeated for 14 generations, until the conclusion of the experiments. Rearings were maintained in an acclimatized room at 23° ± 2°C, 60% ± 10% RH and 12 L:12 D photoperiod.

### Aphid olfactory preference

Aphid olfactory preference was assessed in dual-choice arena assays according to the method described in Santos et al. [49]. The arena consisted of a Petri dish (15 cm diameter) with two rectangular holes in the bottom covered with two layers of fine-mesh fabric to prevent aphids from inserting their stylets into the leaves. Without being excised, a pair of leaves from -Si and +Si wheat plant was positioned below the Petri dish bottom and between the holes and a cardboard support. We conducted eight replicates in darkness in the laboratory under climate-controlled conditions (23° ± 2°C and 60% ± 10% RH). Twenty wingless adult aphids were released in the center of the arena, which was then closed, and the aphids were observed under red light. The number of aphids on the fine-mesh fabric above the leaves was recorded at 10, 30 and 60 min. Aphids were used only once in the dual choice tests.

### Aphid performance

*Rhopalosiphum padi* performance was assessed based on the colony growth in +Si and -Si wheat plants [49]. Each plant was infested with 40 first-instar aphids, selected based on their size from laboratory rearing, and covered by a fine-mesh fabric bag (20 × 30 cm) attached to the pot with an elastic band. Apterae and alate aphids were counted 7 d later. We performed 12 replicates in a room under the environmental conditions used for insect rearing (see above).

### Parasitoid olfactory preference

The response of *L*. *testaceipes* females to volatiles released by wheat plants was assessed in a glass Y-tube olfactometer (side and main arms: 11 cm long × 3 cm Ø). The olfactometer, positioned vertically, was connected to an ARS Volatile Collection System (ARS, Gainesville, FL, USA). Charcoal-filtered, humidified air was pushed through two custom-made glass chambers (2-L capacity), each containing a single wheat plant, and then through the olfactometer side arms at 0.4 l/min/arm. Wasps were introduced singly into the olfactometer main arm and were observed for up to 5 min. A wasp was considered to have made a choice once it crossed a line at 1 cm from the distal end of one side arm and remained in this part for at least 20 s. Wasp that did not respond within 5 min, it was considered non-responsive and was discarded from the statistical analyses. Wasps were tested only once. The sides of the odor sources were reversed after every trial to avoid directional bias. We conducted 30–34 replicates using different plant pairs for each tested parasitoid. After each set of trials, the olfactometer and chambers were washed with detergent (10%), rinsed with distilled water, alcohol (99.5%) and acetate (99.5%), and dried at 160°C for at least 1 h. Olfactometer bioassays were conducted in the photophase, from 14:00 to 17:00 h under climate-controlled conditions (25 ± 1°C and 60% ± 10% RH).

We assessed the olfactory preference of 1- to 2-day-old honey-fed mated *L*. *testaceipes* females, with no experience of oviposition, exposed to plants of the treatments: *(i)* -Si uninfested wheat (-Si), *(ii)* +Si uninfested wheat (+Si), *(iii)* -Si aphid-infested wheat (-Si +Aphid), and *(iv)* +Si aphid-infested wheat (+Si +Aphid) over clean air (only pot with soil). Treatments that were preferred by wasps over clean air were tested against each other. To infest wheat plants with *R*. *padi*, each plant (either -Si or + Si wheat) was infested with 40 third- to fourth-nymphal instar aphids 24 h before assays. To avoid the influence of odors from hosts on the olfactory response of *L*. *testaceipes*, aphids and their exuviae were removed before plants were used in assays.

### Volatile collection and analysis

Six wheat plants per treatment (+Si uninfested, -Si uninfested, +Si aphid-infested and -Si aphid infested wheat plants) were enclosed individually in glass chambers and connected to the ARS Volatile Collection System. Charcoal-filtered, humidified air was pushed into the glass chambers at 1.0 l/min and pulled at same flow rate by a vacuum pump through collectors of volatiles attached to the outlets of chambers. Collectors consisted of a glass pipette with 30 mg of 80/100 mesh Hayesep-Q® adsorbent (Supelco, Bellefonte, PA, USA). Volatiles were collected continuously for 7 h during the photofase (from 09:00 to 16:00). Trapped volatiles were eluted from collectors with 150 μl of dichloromethane containing nonyl acetate (10 ng/μl) as an internal standard into 2-mL glass vials, which were stored at -30°C until analysis. A 2-μl aliquot was injected splitless into a Varian CP-3800 gas chromatograph coupled to a Varian 4000 mass spectrometer fitted with an HP-5MS capillary column (30 m × 0.25 mm i.d. × 0.25 μm film; Agilent, Santa Clara, CA, USA), coupled to a 4000 Ion Trap Mass Spectrometer (Varian, Palo Alto, CA, USA). Helium was used as carrier gas at linear velocity of 1 ml/min. The injector temperature was 250°C and the GC oven was programmed at 40°C for 5 min, increased at 5°C/min to 150°C (held for 1 min), and then increased at 20°C/min to 250°C (held for 20 min). The signal from the detector was processed in the software Workstation version 6.9. Plant volatiles were tentatively identified by comparing their mass spectra with those of the NIST08 library. Ratio of the compounds were calculated based on the peak area relative to that of the internal standard (nonyl acetate).

### Statistical analyses

Data of Si content in wheat leaves, aphid counts in choice preference, and performance were initially tested for normality and homogeneity of variances with the Shapiro-Wilk and Levene tests, respectively. Si relative amounts and data on aphid performance were analyzed by *t*-test. Data on aphid preference along time course was analyzed by a general linear mixed model (GLMM, treatment as a fixed effect and time as a random effect) with Laplace approximation and Poisson distribution. Parasitoid choice in Y-tube bioassays was evaluated using chi-square test. Plant volatile composition values were analyzed by multivariate analysis of variance (MANOVA) and principal component analysis (PCA). Total volatile amounts emitted by the uninfested and aphid-infested plants were analyzed by a log-linear model based on Poisson distribution with overdispersion, followed by Tukey post-hoc test. To test the effect of Si treatment on the emission of individual compounds by uninfested or aphid-infested plants, data on relative quantities was analyzed by general linear model (GLM) and goodness-of-fit test was performed to select the best-fit model for the emission of each compound. Statistical analyses were carried out by using the R statistical software (version 3.6.2) with the interface R.

## Results

### Si supplementation

Leaves of +Si wheat plants accumulated about 1.8-fold more Si content (0.77 ± 0.55%) than -Si plants (0.42 ± 0.06%) (*t* = 3.74, *P* = 0.003)

### Aphid preference and performance

**Fig 1:**
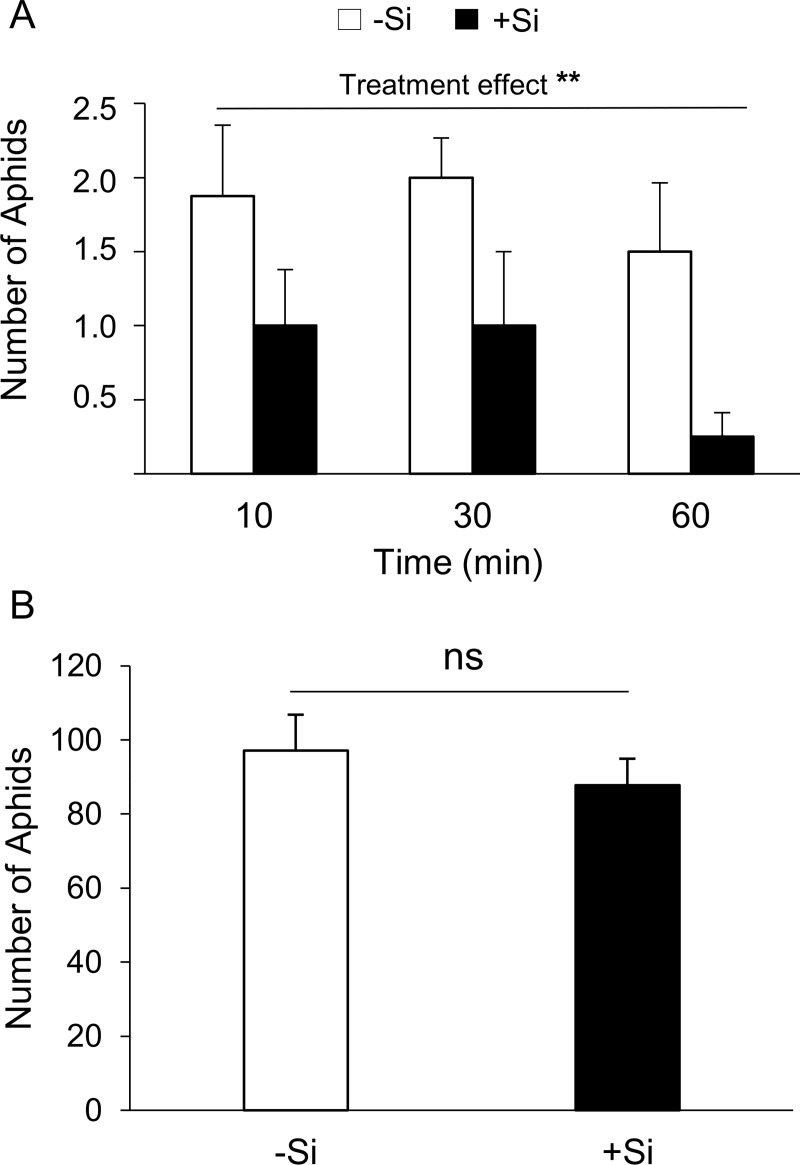
Effect of silicon supplementation on wheat direct defenses to the bird cherry-oat aphid. (A) Olfactory preference of *Rhopalosiphum padi* apterae (mean number of aphids ± SE) to Si-supplemented (+Si) and non-Si supplemented (-Si) wheat plants in arena choice assays along time course; (B) *R*. *padi* performance in +Si and -Si wheat plants based on the total number of aphids (mean number of aphids ± SE). ns = not significant; ** indicates significant difference between treatments at 1% according to GLMM.

Aphids, in dual-choice tests, preferred volatiles emitted from -Si wheat plants over +Si wheat plants (Fig 1A, GLMM, treatment effect: χ^2^ = 9.67, *P* = 0.002). On average, we observed about 6 times more aphids on the area with odors emitted by -Si wheat plants than +Si plants. Nevertheless, in the aphid performance assay, aphid colony size was similar in -Si and +Si wheat plants (Fig 1B, *t* test, *P* > 0.05).

### Parasitoid olfactory preference

**Fig 2:**
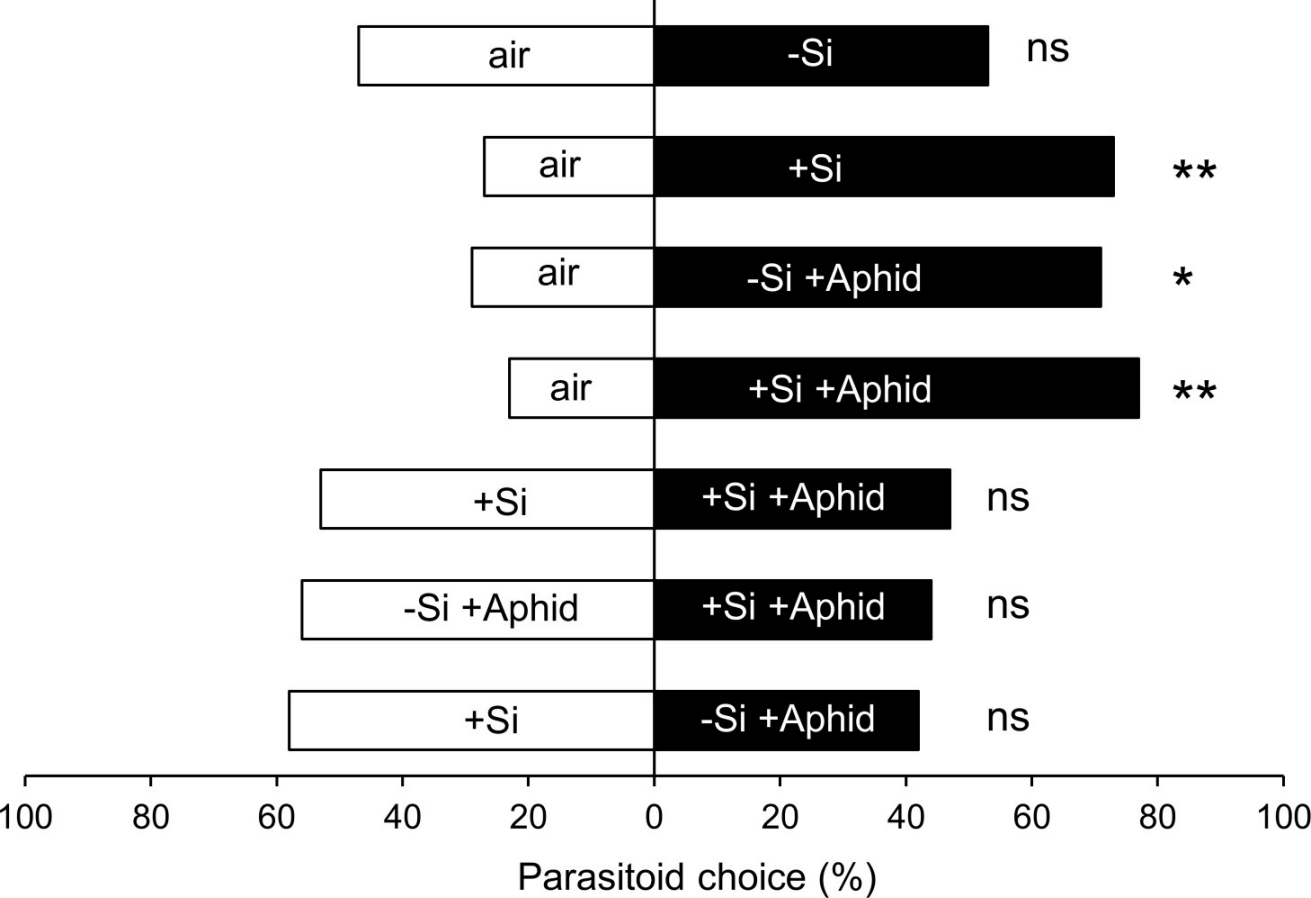
Effect of silicon supplementation on the olfactory response of *Lysiphlebus testaceipes* to wheat volatiles. Olfactory response of the aphid parasitoid *Lysiphlebus testaceipes* to volatile emissions from Si-supplemented (+Si) and non-Si supplemented (-Si) uninfested (-aphid) and aphid-infested (+aphid) wheat plants, in the Y-tube olfactometer. The olfactory response of parasitoid females to the treatments were first tested against the clean air (air). Treatments that were attractive were contrasted among themselves. * indicates significant difference at 5% according to chi-square test. ** indicates significant difference at 1% according to chi-square test.

Female *L*. *testaceipes* parasitoids, exposed to wheat plant volatiles *vs*. clean air, were attracted to volatiles emitted from +Si uninfested, -Si-aphid-infested and +Si aphid-infested wheat plants (binomial test, clean air *vs*. +Si: χ^2^ = 6.53, *P* = 0.010; clean air *vs*.–Si +Aphid: χ^2^ = 6.12, *P* = 0.013; clean air *vs*. +Si +Aphid: χ^2^ = 8.53, *P* < 0.01), but not to those from -Si uninfested wheat plant (Fig 2, *P* > 0.05). Wasps did not discriminate between +Si uninfested from +Si aphid-infested wheat plants, -Si aphid-infested from +Si aphid-infested wheat plants, or +Si uninfested from -Si aphid-infested wheat plants (Fig 2, *P* > 0.05).

### Volatile emission

Analysis of the volatile composition revealed significant differences among treatments (Table 1, MANOVA, *F*_*3*,*27*_ = 2.24, *P* = 0.018). PCA showed separation between uninfested and aphid-infested treatments along PC1, which aggregated 63% of variance, but not between +Si and -Si treatments (S1 Fig). -Si and +Si aphid-infested plants released greater total relative amounts of volatiles compared to -Si and +Si uninfested wheat plants (Table 1, GLM, χ^2^ = 60.07, *P* < 0.001, Tukey’s test *P* < 0.05). Although not statistically significant, +Si uninfested plant released about 3.5-fold more volatiles than -Si uninfested plants. Taken individually, geranyl acetone was the only compound being released at higher concentrations by +Si uninfested relative to emission of -Si uninfested plants (Table 1, GLM, *t* = 2.04, *P* < 0.05) and similar to that emitted by +Si aphid-infested plant (Table 1, GLM, *t* = 0.33, *P* = 0.749). Aphid herbivory in -Si wheat plants induced up-regulation of all compounds and emission of two novel volatiles (3-decen-5-one and methyl geranate), but the blend was qualitatively and quantitatively similar blend to that emitted by +Si aphid-infested plants.

**Table 1:** Ratio of compounds (mean ± SE) emitted by silicon-supplemented (+Si) and non-silicon supplemented (Si-) uninfested and aphid-infested wheat plants. Relative amounts were estimated based on the peak area of the internal standard. Bold value indicates significant elevation in relative amount of a compound compared to the control treatment (-Si uninfested wheat plant) according to GLM.

Compounds	Uninfested		Aphid-infested	
	-Si	+Si	-Si	+Si
linalool oxide	0.35 ± 0.06	0.49 ± 0.12	4.49 ± 1.26	4.14 ± 0.94
benzaldehyde	0.18 ± 0.03	0.26 ± 0.05	1.16 ± 0.21	0.83 ± 0.40
undentified monoterpene 1	0.28 ± 0.17	0.81 ± 0.49	8.47 ± 4.76	11.09 ± 2.34
undentified monoterpene 2	0.22 ± 0.11	0.21 ± 0.03	19.50 ± 11.63	19.36 ± 7.90
3-decen-5-one	-	-	9.36 ± 2.51	9.68 ± 1.19
(*E*)-2-dodecen-1-ol	0.11 ± 0.01	0.28 ± 0.10	1.99 ± 0.42	3.13 ± 0.92
methyl geranate	-	-	5.22 ± 2.28	7.08 ± 2.59
Geranyl acetone	0.42 ± 0.17	**3.66 ± 1.72**	3.43 ± 0.89	4.41 ± 1.19
Total Emission ^a^	1.57 ± 0.37 a	5.73 ± 2.19 a	53.62 ± 15.25 b	59.72 ± 12.56 b

^a^ different letters indicate significant differences according to Tukey’s test at 5% probability

## Discussion

As we have gained more insight into the underlying mechanisms that account for the enhanced resistance of Si-supplemented plants to herbivores, studies have focused on addressing the effects of Si accumulation via defense signal transduction pathways [22, 50]. Studies on rice indicate that the physical barrier formed by Si accumulation makes plants more resistant to herbivores before their arrival and, once Si-supplemented plants are upon attack, greater levels of induced defenses results from priming of JA-modulated antiherbivore defenses [11, 12, 22, 29]. Unlike rice, the results presented here indicate that Si accumulation in wheat did not work as a priming agent, but as an elicitor of chemical defenses as +Si uninfested plants became more resistant, but not those infested by aphids.

Prior studies showed that uninfested grasses grown in Si supplemented soil is less preferred hosts for aphids than plants grown in non-Si supplemented soil [35, 44, 51, 52] likely because of chemical induced defenses as the mechanical resistance seems not to play an important role in this interaction [34, 37]. Here, by testing the olfactory response of the bird cherry-oat aphid *R*. *padi* to +Si wheat plant volatile emission, we found that volatile chemicals are involved in Si-induced resistance. Number of aphids was about 6-fold higher on the odor field from -Si than +Si wheat plants, suggesting a non-preference behavior. Comparison between volatile profiles emitted by -Si and +Si uninfested wheat plants revealed that only a single compound, the terpene geranyl acetone, was found at higher amounts in +Si plant volatile emissions. Molecules of the geranyl group potentially function as analogs of (*E*)-beta-farnesene (EBF), a common molecule of aphid alarm pheromone that is also shared by *R*. *padi* [53], because of their affinity to the same odorant-binding protein [54, 55]. Although far more studies investigated the activity of geranyl acetate to aphids [55–57], repellency of geranyl acetone to the green peach aphid *Myzus persicae* (Sulzer) (Hemiptera: Aphididae) has been recently demonstrated [58, 59]. Therefore, the augmented concentration of geranyl acetone in the volatile emission might mediate the non-preference behavior of the bird cherry-oat aphid *R*. *padi* to +Si uninfested wheat plants. Future studies should confirm the repellency of geranyl acetone to *R*. *padi* and whether it is due to the molecule affinity to the odorant-binding protein that binds with EBF, as demonstrated for geranyl acetate.

Despite that +Si uninfested wheat plants were less preferred by the aphids, Si accumulation in wheat plant tissue did not impact *R*. *padi* colony growth. Although aphid performance was assessed in a short-term experiment, our result corroborates with recent and detailed studies demonstrating no effect of Si-supplemented wheat and corn on *Rhopalosiphum maidis* (Fitch) (Hemiptera: Aphididae) biology and feeding behaviour [44, 60]. Despite that Si soil supplementation in wheat seems not effective on reducing aphid performance, it reduces host preference by aphids [21, 34, 35]. Therefore, taken together, our study and the literature suggest that Si uptake and accumulation in wheat induce a non-preference type of resistance to aphids mediated by plant volatiles.

While wheat volatile emission induced by Si supplementation was non-preferred by aphids, it was attractive to its parasitoid *L*. *testaceipes*. Again, geranyl acetone, the only up-regulated compound by Si supplementation, is the potential candidate for mediating the attraction of the parasitoid to +Si uninfested plants. Aphid herbivory elicited the release of a couple of exclusive compounds, but mostly increased the concentrations of compounds that were present in the constitutive blend. Even though aphid infestation in either–Si or +Si induced the release of attractive blends to the parasitoid, Si supplementation did not enhance the attractiveness of herbivore-induced plant volatiles to *L*. *testaceipes*, as we first predicted. The fact that Si accumulation did not alter the composition of aphid-induced plant volatiles explains the lack of discrimination of the parasitoid between +Si and–Si aphid-infested plants. Interestingly, emission of +Si uninfested plant was as attractive as +Si aphid-infested plant or -Si aphid-infested plant to the parasitoid. This result gives support to the hypothesis that geranyl acetone is responsible for the enhanced attraction of +Si uninfested wheat to the parasitoid since the terpene is released at similar concentrations by the three treatments. Up to date, there is no report on the activity of geranyl acetone to aphid parasitoids in the literature but, as a potential analogue of EBF, which is attractive to aphid natural enemies [61], it may also function as an attractant. For example, the odorant-binding protein responsible for EBF reception in the lacewing, a natural predator of aphids, also binds with structurally similar molecules, such as geranyl acetone and geranyl acetate [62, 63]. Given that *L*. *testaceipes* is also a generalist aphid parasitoid that is attracted to cornicle secretions of *R*. *padi*, which contains EBF [54, 64], it seems plausible that geranyl acetone is attractive to the parasitoid, but further investigation is needed to confirm this hypothesis.

Given the parasitoid’s learning ability, the attraction of the parasitoids to non-rewarding odors, such as those emitted by +Si uninfested wheat plants that mistakenly guide them to plants with no hosts, can negatively affect its foraging efficiency [65, 66]. Unlike previous studies in which Si supplementation increases the attractiveness of HIPV blends to natural enemies [29, 32], our study revealed that Si supplementation by itself elicits in wheat the release of attractive volatile blend to the parasitoid, raising concerns about the implementation of this tactic for integrating with biological control.

Overall, our results indicate that Si supplementation in wheat has potential to control the bird cherry-oat aphid population because Si accumulation in plants make them less likely to be colonized by the bird cherry-oat aphid at the same time that recruits its parasitoid *L*. *testaceipes*. Our data strongly suggest that the constitutive increased concentration of geranyl acetone, a potential mimic of EBF, released by +Si wheat commonly mediates those interactions. In a similar way, genetically-transformed wheat constitutively emitting EBF repels aphids and attracts their natural enemies under controlled conditions [67]. Nevertheless, in the field, transformed wheat did not result in control of aphid population likely because of aphid habituation to EBF [67]. In our study system, future studies should address whether: *(i)* constitutive release of geranyl acetone causes habituation in aphids; and *(ii)* the parasitoid’s attraction to +Si uninfested and lack of discrimination between odors of +Si uninfested and aphid-infested treatments lead to negative consequences to host finding.

## Supporting information

S1 Fig Volatile emission by +Si and -Si uninfested plants differ from those emitted by +Si and -Si aphid-infested plants. Loading plot of Principal Component Analysis (PCA) performed on concentrations of volatile compounds in the blend emitted by non-Si supplemented uninfested (-Si) and aphid-infested (-Si +Aphid), Si-supplemented uninfested (+Si) and aphid infested (+Si +Aphid).
(DOCX)
