# Supplementary material for: Silicon-induced changes in plant volatiles reduce attractiveness of wheat to the bird cherry-oat aphid Rhopalosiphum padi and attract the parasitoid Lysiphlebus testaceipes
Source: PLoS One. 2020 Apr 3;15(4):e0231005. doi: 10.1371/journal.pone.0231005 (PMC7122784; doi:10.1371/journal.pone.0231005)
Supplement: S1 Fig — Loading plot of Principal Component Analysis (PCA) performed on concentrations of volatile compounds in the blend emitted by non-Si supplemented uninfested (-Si) and aphid-infested (-Si +Aphid), Si-supplemented uninfested (+Si) and aphid infested (+Si +Aphid). (DOCX) [file pone.0231005.s001.docx]

**S1 Fig. Volatile emission by +Si and -Si uninfested plants differ from those emitted by +Si and -Si aphid-infested plants.** Loading plot of Principal Component Analysis (PCA) performed on concentrations of volatile compounds in the blend emitted by non-Si supplemented uninfested (-Si) and aphid-infested (-Si +Aphid), Si-supplemented uninfested (+Si) and aphid infested (+Si +Aphid).
